# Biochemical Properties of Enzymes Present in Microsomal Fractions of Arabidopsis Leaves Involved in Synthesis of Esters of Free Fatty Acids with Alcohols of Different Chain Lengths

**DOI:** 10.3390/ijms27125211

**Published:** 2026-06-09

**Authors:** Alicja Czyż, Katarzyna Jasieniecka-Gazarkiewicz, Antoni Banaś

**Affiliations:** Intercollegiate Faculty of Biotechnology, University of Gdańsk and Medical University of Gdańsk, 80-307 Gdańsk, Poland

**Keywords:** wax esters, fatty acids ethyl esters, phytol, free fatty acids, membranes

## Abstract

In the performed studies, microsomal fractions from Arabidopsis plants were used as a source of enzymes that esterify free fatty acids to free alcohols (both fatty alcohols and short-chain alcohols). The existence of two isoforms of tested enzymes is postulated. These putative isoforms, characterized by apparent peak activities at pH 6.0 and pH 7.2, respectively, appeared to differ in their responses to environmental conditions, e.g., in their temperature dependence; in their responses to calcium and magnesium ions’ presence in assays; or in retaining activity after membrane solubilization with 4% detergent CHAPS (only the postulated isoforms most active at pH 6.0 kept their activity). Differences were also annotated in the preferences of the apparent enzymatic activities toward different fatty alcohols (although both putative isoforms showed the highest activity toward phytol) and different fatty acids (in reactions with both fatty alcohols and short-chain alcohols). Two apparent enzymatic activities were inhibited by tetrahydrolipstatin, which may suggest their lipase nature. Based on the high activity of the tested enzymes in the membrane of the microsomal fractions, the authors speculate that their physiological function might be associated with maintaining membrane integrity against the harmful effects of excess free fatty acids and/or free fatty alcohols.

## 1. Introduction

In plants, fatty acids are synthesized de novo exclusively in chloroplasts by enzymes of the fatty acid synthase complex. After synthesis, they can be utilized for chloroplast lipid biosynthesis or transported to the cytosol in the form of acyl-CoA. In the cytosol, they can be directly utilized for the biosynthesis of extraplastidic lipids or first be subjected to an elongation process. Further modification of their structure occurs after incorporation into lipids, especially phosphatidylcholine [1,2]. In complex lipids, fatty acids are esterified either to glycerol (e.g., phospholipids, glycolipids, diacylglycerols, triacylglycerols) or to sphingolipid bases (sphingolipids). They can also be esterified to primary alcohols. Among the last group, wax esters (esters of a fatty alcohol and a fatty acid) are the main class [1]. In plants, wax esters can be found mainly in epicuticular waxes, a hydrophobic layer covering the cuticle. The main role of this layer is the protection of plant organs (e.g., leaves, fruits) against water loss, pathogens, insect attacks, and UV light [1,3,4]. In jojoba seeds, wax esters serve as storage materials [5,6]. They play a similar role in many pelagic marine organisms, especially in cold, deep-water environments [7]. Wax esters are also constituents of mammalian sebum and meibum [8]. Knowledge regarding fatty acids esters with low-molecular-weight alcohols is much more limited. However, such esters have been found in plant extracts. Fatty acid methyl esters have been found, for instance, in the pollen of *Zea mays* [9] and in the oils of walnuts, peanuts, almonds, soybeans and filberts [10]. In the fruit arils and seeds of four *Euonymus* species (*Euonymus* sp.), 19 fatty acid lower-alkyl esters have been identified, including fatty acid methyl ester (FAMEs), fatty acid ethyl esters (FAEEs), fatty acids propyl esters (FAPEs), and fatty acids *n*-butyl esters (FABEs) [11]. A rich source of such fatty acid esters (including FAMEs and FAEEs) can be found in the leaves of the medicinal plant *Alstonia boonei* [12]. Fatty acid methyl and ethyl esters have also been identified in extracts of the green microalga *Chlamydomonas reinhardtii* [13]. *Saccharomyces cerevisiae* and many other fungi produce a range of fatty acid ethyl esters, especially those of medium chain length [14,15].

The best-known enzymes involved in the synthesis of wax esters are acyl-CoA: fatty alcohol acyltransferases (wax synthases, WS). The genes encoding WS have been cloned from microorganisms, animals, and plants, and the enzymes encoded by these genes are well characterized [16,17,18]. The esterification of free fatty acids with free fatty alcohols has also been reported. Kolattukudy [19] presented data showing that acetone powder of broccoli leaves contains enzymes that can use free fatty acids, fatty acyl-CoA, or fatty acids esterified in triacylglycerol molecules for esterification of fatty alcohols. The presence of enzymes catalyzing the esterification of free fatty acids and free fatty alcohols has also been shown in microsomal fractions of Arabidopsis leaves [20] and microsomal fractions of wheat roots [21]. The enzyme/s catalyzing such reactions have not been identified; however, it has been shown that they may contain a lipase motif, as tetrahydrolipstatin (a well-known lipase inhibitor) efficiently inhibited their activity [20,21]. The ability of plant lipases to synthesize wax esters from free fatty acids and free fatty alcohols was recently shown [6].

Fatty acid ethyl esters are produced in microorganisms by direct enzymatic esterification of ethanol with carboxylic acids (catalyzed by FAEE synthases/carboxylesterases) or by the transfer of an acyl group from acyl-CoA to alcohols, catalyzed by acyl-CoA: ethanol O-acyltransferases (AEATases) [22]. In the yeast *S. cerevisiae*, two genes encoding enzymes with AEATase activity (which preferentially esterify medium-chain fatty acids) have been identified [15]. In mammalian tissues, both AEATases and FAEE synthase activities have been demonstrated. Enzymes possessing AEATase activity have not yet been identified, whereas enzymes with FAEES activity have been purified from several animal tissues [23]. In plants, the enzymes involved in fatty acid ethyl ester synthesis have not yet been identified. However, FAEE synthase-type enzymatic activity has been demonstrated in experiments with microsomal fractions of Arabidopsis leaves and microsomal fractions of wheat roots [20,21]. In assays with these microsomal fractions, free fatty acids were efficiently esterified with ethanol without prior activation of the fatty acids to acyl-CoA. A similar type of activity was also shown in assays with microsomal fractions from the yeast *S. cerevisiae* [20]. The synthesis of fatty acid ethyl esters in these assays was strongly inhibited by tetrahydrolipstatin, suggesting that the enzymes catalyzing these reactions could be of the lipase type. Recently, *Candida rugosa* lipase was also shown to efficiently synthesize FAEEs using free fatty acids and ethanol as substrates [24].

The present work aimed to shed more light on the biochemical properties of enzymes catalyzing the esterification of free fatty acids with long-chain fatty alcohols, as well as with lower-molecular-weight alcohols, especially ethanol. The effects of various environmental factors, including pH, temperature, and the presence of divalent cations, together with substrate specificity towards different fatty acids, fatty alcohols, and low-molecular-weight alcohols, were studied. Microsomal fractions isolated from Arabidopsis plants were used as the enzyme source.

## 2. Results

### 2.1. Biochemical Properties of Enzymes in Microsomal Fraction of Arabidopsis Leaves Capable of Synthesizing Wax Esters and Fatty Acid Ethyl Esters

#### 2.1.1. Dependency of Fatty Acid Ethyl Ester and Wax Ester Synthesis on Microsomal Fraction Concentration

**Figure 1:**
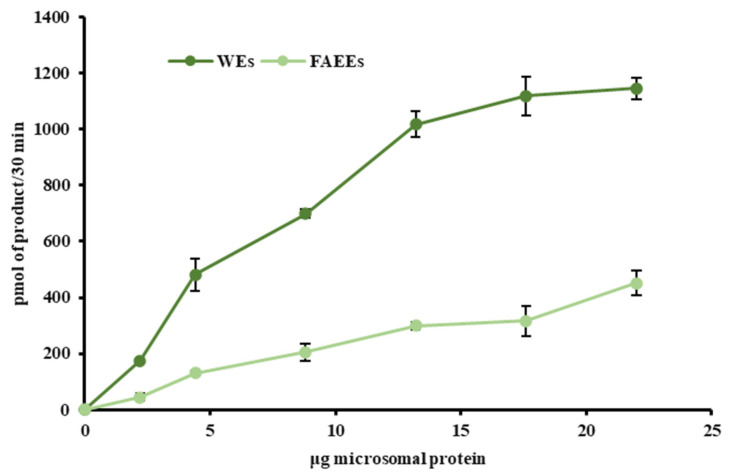
Effect of microsomal protein concentration on the synthesis of wax esters (WEs) and fatty acid ethyl esters (FAEEs). The assays were performed using increasing amounts of Arabidopsis leaf microsomal protein in HEPES buffer (pH 7.2) at 30 °C for 30 min. Data are presented as the mean ± SD from at least three replicates obtained from one microsomal preparation isolated from 10 to 15 plants.

In our previous work [20], HEPES buffer (50 mM, pH 7.2) containing 10 mM MgCl_2_ was used in assays to characterize the enzymes under study. However, in preliminary studies we have found that Mg^2+^ ions present in the incubation buffer strongly reduced the activity of the tested enzymes (more detail is provided in the chapter concerning the effects of cations). Thus, in subsequent experiments, buffers without added ions were used. The characterization of the tested enzymes began with optimization of the amount of microsomal fraction used in the assays. Aliquots of microsomes containing from 2.2 to 22 µg of protein were tested. An increase in the concentration of the microsomal fraction resulted in higher amounts of both synthesized wax esters (WEs) and fatty acid ethyl esters (FAEEs). However, the increase in the amount of synthesized products, especially WEs, was not proportional to the increase in membranes above 13.2 µg of membrane protein per assay (Figure 1). Therefore, aliquots of microsomes containing 13.2 µg of membrane protein were used in all subsequent assays.

#### 2.1.2. Effect of pH on the Efficiency of Fatty Acid Ethyl Esters and Wax Esters Biosynthesis

**Figure 2:**
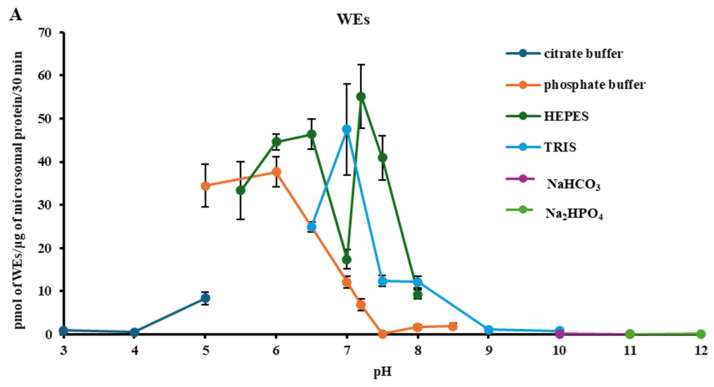

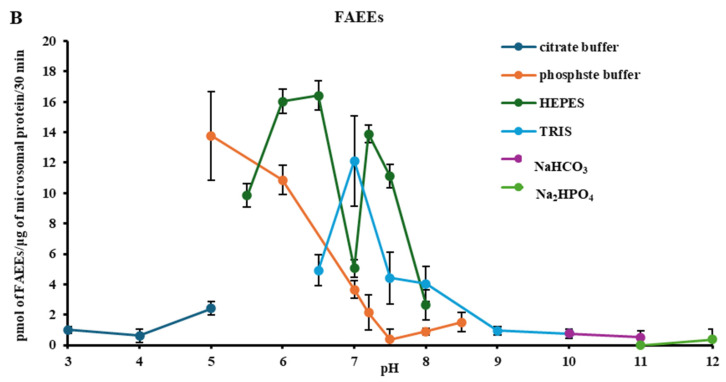
Effects of pH and buffer type on the efficiency of the synthesis of wax esters (**A**) and fatty acid ethyl esters (**B**) in assays with microsomal fractions of Arabidopsis leaves. Assays were performed using microsomal fractions containing 13.2 µg of protein in various buffer systems (50 mM): citrate (pH 3–5), phosphate (pH 5–8.5), HEPES (pH 5.5–8.0), Tris-HCl (pH 6.5–10), NaHCO_3_ (pH 10–11), and Na_2_HPO_4_ (pH 11–12). The reactions were carried out for 30 min at 30 °C. Data are presented as the mean ± SD from at least three replicates obtained from one microsomal preparation isolated from 10 to 15 plants.

Six different 50 mM incubation buffers were used in the experiments: citrate buffer (pH 3–5), phosphate buffer (pH 5–8.5), HEPES buffer (pH 5.5–8), Tris-HCl buffer (pH 6.5–10), NaHCO_3_ buffer (pH 10–11) and Na_2_HPO_4_ buffer (pH 11–12). The tested enzymatic activity depended on both the pH of the environment and the type of buffer used. Generally, the efficiency of WE and FAEE synthesis was low at both acidic (pH 3–4) and alkaline pH (pH 9–12). The highest activity of the tested enzymes was recorded within the pH range of 5–8. However, the type of incubation buffer strongly affected this activity. In assays performed with HEPES buffer, two activity peaks were observed: one at pH 6–6.5 and another at 7.2–7.5. When phosphate buffer was used, only one activity peak, between pH 5 and 6, was observed. In a more alkaline environment, the activity declined, reaching a minimum at pH 7.5. This peak likely corresponds to the first activity peak observed in HEPES buffer assays. In contrast, in assays with Tris-HCl buffer, only the peak corresponding to the second peak noted in HEPES assays was recorded. However, Tris-HCl buffer shifted this maximum towards pH 7.0. The first peak of activity was not observed because the lowest pH tested with this buffer was 6.5; therefore, the first peak could have been shifted towards a more acidic pH range. The effects of pH on enzymatic activity were similar for both FAEE and WE synthesis (Figure 2A,B). The presence of two activity peaks may indicate the existence of two enzymes or enzyme isoforms with the ability to synthesize FAEEs and WEs. Therefore, subsequent experiments were performed using HEPES buffer at both pH 6.0 and pH 7.2.

#### 2.1.3. Dependency of the Synthesis of Wax Esters (WEs) and Fatty Acid Ethyl Esters (FAEEs) on the Reaction Time

**Figure 3:**
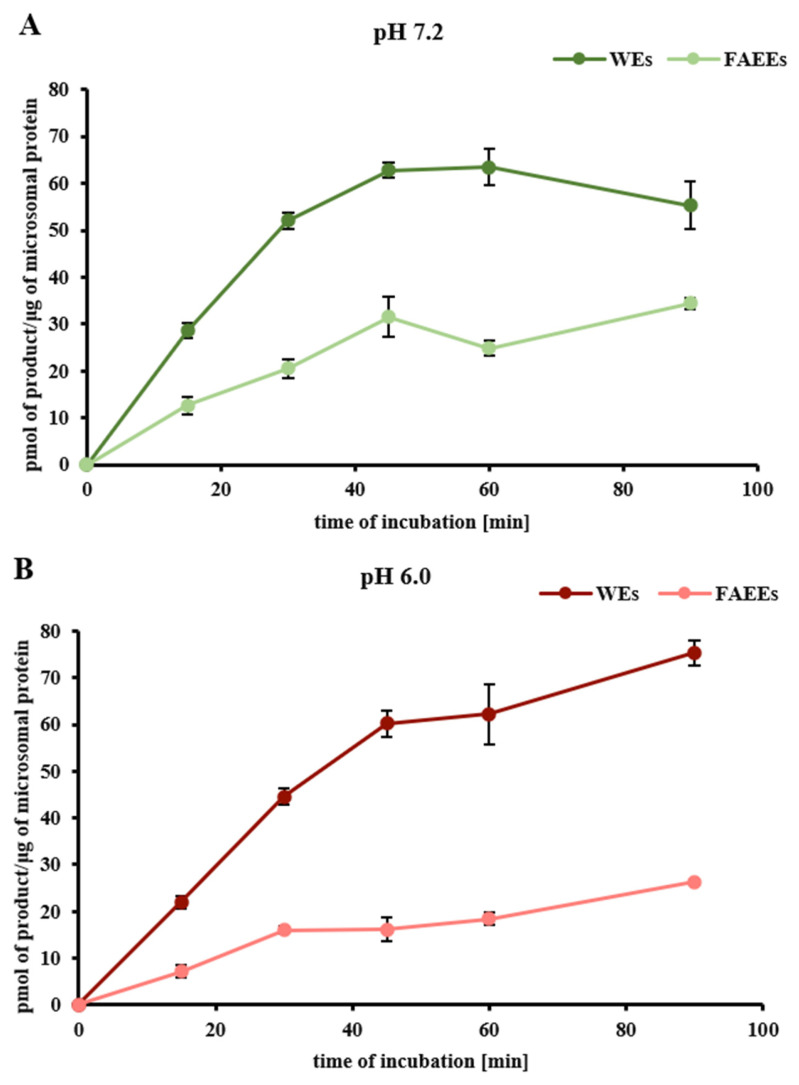
Time course of wax ester (WE) and fatty acid ethyl ester (FAEE) synthesis in Arabidopsis leaf microsomes at pH 7.2 (**A**) and pH 6.0 (**B**). Assays were performed using microsomal fractions containing 13.2 µg protein in HEPES buffer at 30 °C. Data are presented as the mean ± SD from at least three replicates obtained from one microsomal preparation isolated from 10 to 15 plants.

In the assays, 50 mM HEPES buffer at pH 7.2 and pH 6.0 was used. In assays with the first buffer, the synthesis of both WEs and FAEEs proceeded in an almost linear manner up to 45 min of reaction time. However, in the case of WEs, the reaction intensity between 30 and 45 min was somewhat lower than during the first 30 min of incubation. The net increase in synthesized WEs between 45 and 60 min was not observed, and after this time, a slight decrease in the amount of produced WEs was noted. This suggests that hydrolytic processes might occur alongside WE synthesis. In assays with HEPES buffer at pH 6.0, WE synthesis proceeded linearly for up to 45 min of incubation. After this period, the rate of WE synthesis decreased; however, a net increase in the amount of produced WEs was recorded until the end of the incubation time (90 min). FAEE synthesis was linear only up to 30 min of reaction and subsequently proceeded in a non-linear manner (Figure 3). The ratio of produced WEs to FAEEs in assays at pH 7.2 was lower than in assays at pH 6.0 (after 45 min of incubation, for the ratio were 2 and 3.7, respectively). These differences in the intensity of WE and FAEE synthesis at the two tested pH values may suggest the existence of potentially distinct enzyme isoforms capable of synthesizing these esters. For further assays, an incubation time of 30 min was selected, as all tested enzymatic activities proceeded linearly during this period.

#### 2.1.4. Effects of Temperature on the Efficiency of Wax Ester (WE) and Fatty Acid Ethyl Ester (FAEE) Synthesis

**Figure 4:**
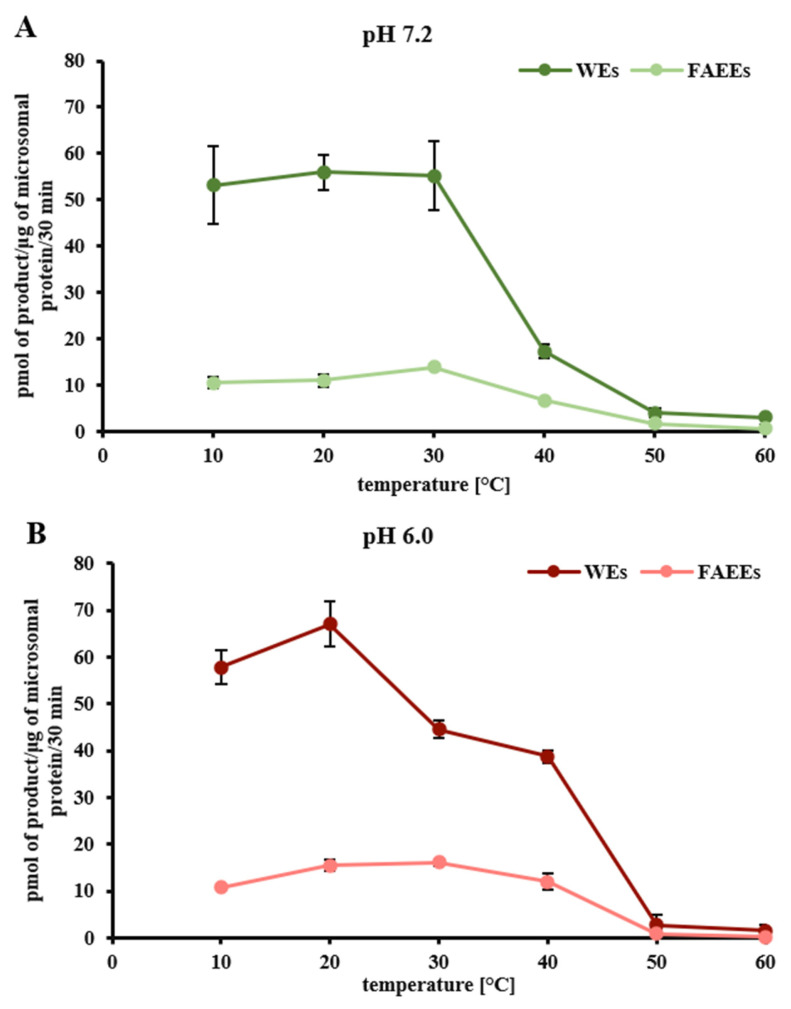
Temperature dependency of the formation of wax esters (WEs) and fatty acid ethyl esters (FAEEs) in assays with HEPES buffer with pH 7.2 (**A**) and with pH 6.0 (**B**) by enzymes of microsomal fractions from Arabidopsis leaves (containing 13.2 µg of protein). Data are presented as the mean ± SD from at least three replicates obtained from one microsomal preparation isolated from 10 to 15 plants.

Clear differences in the temperature dependence of WE and FAEE synthesis intensity were observed between assays performed at pH 7.2 and those conducted at pH 6.0. In the first case, increasing the temperature from 10 to 30 °C only slightly enhanced the synthesis intensity of both types of esters; however, this increase was more pronounced for FAEE synthesis. A further increase in temperature from 30 to 40 °C significantly reduced the amount of synthesized products, with a more drastic effect observed for WE synthesis. At 50 °C, both enzymatic activities were very low, close to the detection limit (Figure 4A). In assays carried out at pH 6.0, WE synthesis activity reached its maximum at 20 °C, representing an approximately 16% increase compared to assays conducted at 10 °C. A further increase in temperature to 40 °C caused a gradual decline to about 60% of maximum recorded activity, followed by a sharp decrease; at 50 °C, WE synthesis was close to the detection limit. FAEE synthesis did not exhibit a similar temperature dependence. Increasing the temperature from 10 to 30 °C slightly enhanced FAEE synthesis, whereas a further increase to 40 °C resulted in a slight decrease in activity. Raising the temperature to 50 °C almost completely inhibited this synthesis (Figure 4B). These results suggest that WE and FAEE synthesis may be catalyzed by distinct enzymes or isoforms, exhibiting the highest activities at either pH 7.2 or pH 6.0. For further assays, a temperature of 30 °C was selected, as most of the tested enzymatic activities were highest at this temperature, except for WE synthesis at pH 6.0 which was approximately 36% lower than the maximum activity recorded at 20 °C.

#### 2.1.5. Effect of Various Cations on the Synthesis Efficiency of Wax Esters (WEs) and Fatty Acid Ethyl Esters (FAEEs)

**Figure 5:**
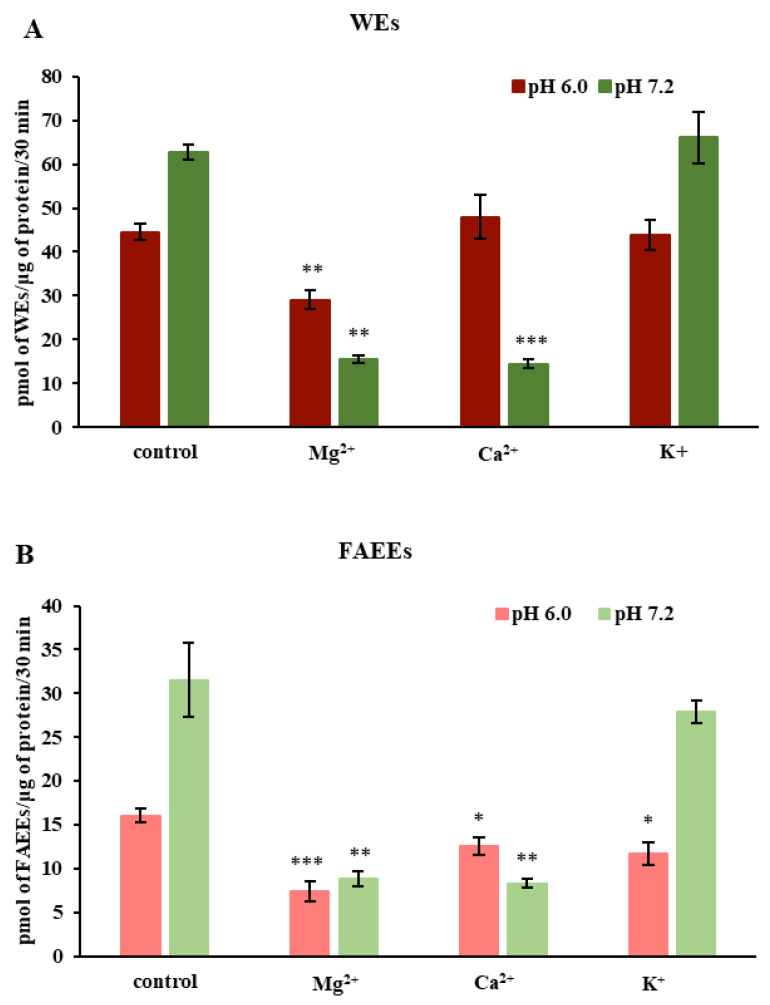
Effect of 10 mM Mg^2+^, Ca^2+^ and K^+^ ions on the formation of wax esters (**A**) and fatty acid ethyl esters (**B**) in HEPES buffer at pH 7.2 and pH 6.0 by enzymes of microsomal fractions from Arabidopsis leaves (containing 13.2 µg of protein). The reactions were carried out for 30 min at 30 °C. Data are presented as the mean ± SD from at least three replicates obtained from one microsomal preparation isolated from 10 to 15 plants. Statistical significance was determined using a two-tailed Student’s *t*-test comparing ion-treated samples with the control (buffer without added ions). Asterisks indicate significant differences: * *p* ≤ 0.05; ** *p* ≤ 0.01; *** *p* ≤ 0.001.

The effects of Mg^2+^, Ca^2+^ and K^+^ ions added to the incubation buffers as chloride salts were studied. Assays were performed at pH 7.2 and pH 6.0. At both pH values, magnesium ions (Mg^2+^) exhibited an inhibitory effect on WE and FAEE synthesis; however, at pH 7.2, the inhibitory effect was more pronounced for FAEE synthesis. Calcium ions (Ca^2+^) inhibited FAEE synthesis under both pH conditions, while WE synthesis was inhibited only at pH 7.2. Potassium ions (K^+^) slightly inhibited FAEE synthesis at both pH values but did not affect WE synthesis (Figure 5). These results support our earlier assumption that WE and FAEE synthesis may be carried out by different enzymes present in microsomal fractions of Arabidopsis leaves. In subsequent assays, HEPES buffer without added ions was used.

#### 2.1.6. Effect of Tetrahydrolipstatin on the Synthesis Efficiency of Wax Esters (WEs) and Fatty Acid Ethyl Esters (FAEEs)

**Figure 6:**
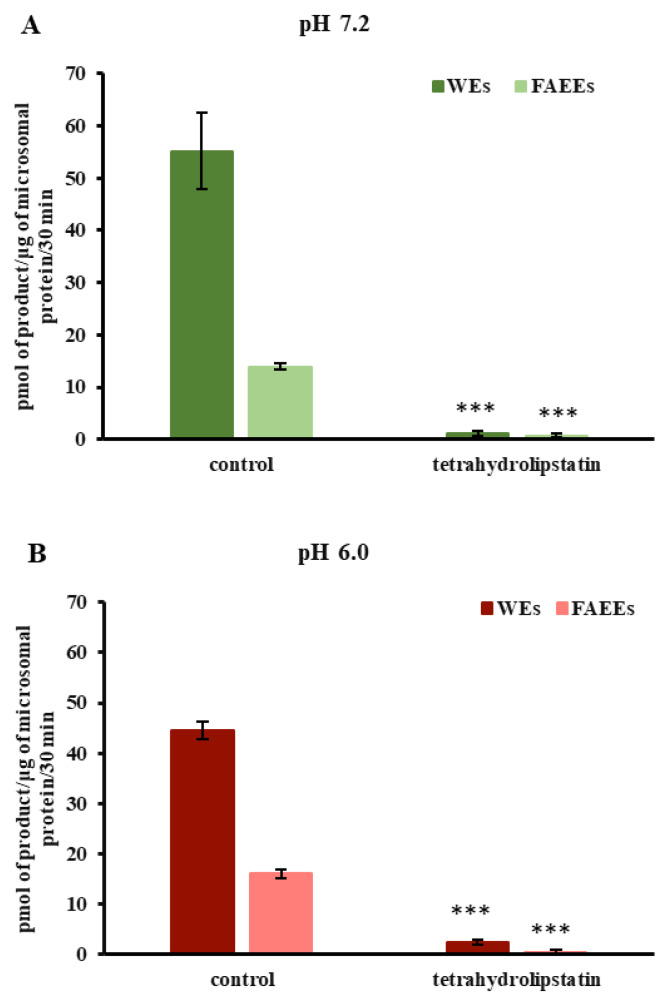
Effect of 20 µM tetrahydrolipstatin on the formation of wax esters (WEs) and fatty acid ethyl esters (FAEEs) at pH 7.2 (**A**) and pH 6.0 (**B**) in HEPES buffer by enzymes of microsomal fractions from Arabidopsis leaves (containing 13.2 µg of protein). The reactions were carried out for 30 min at 30 °C. Data are presented as the mean ± SD from at least three replicates obtained from one microsomal preparation isolated from 10 to 15 plants. Statistical significance was determined using a two-tailed Student’s *t*-test comparing tetrahydrolipstatin-treated samples with the untreated control. Asterisks indicate significant differences: *** *p* ≤ 0.001.

Tetrahydrolipstatin is a well-known inhibitor of lipases [6]. Previous studies on enzymes exhibiting activities similar to those investigated in the present study demonstrated that this compound efficiently inhibited FAEE and WE synthesis from free fatty acids and free ethanol or free long-chain fatty alcohols [20]. Here, we tested its effect on FAEE and WE synthesis at two different pH values: 7.2 and 6.0. Under control conditions, the efficiency of WE synthesis was higher than that of FAEE synthesis, consistent with our earlier findings. Similarly to the previous results, the ratio of formed WEs to FAEEs (nmol of formed WEs/nmols of formed FAEEs) was higher at pH 7.2 (approximately 4.0) than at pH 6.0 (approximately 2.9). The addition of tetrahydrolipstatin at a concentration of 20 µM efficiently inhibited the synthesis of both WEs and FAEEs. In assays carried out at pH 7.2, WE synthesis was inhibited by approximately 98%, whereas FAEE synthesis was inhibited by about 94%. In contrast, at pH 6.0, the inhibition of FAEE synthesis was higher (about 98%) than that of WE synthesis (accounting for approximately 93%) (Figure 6).

#### 2.1.7. Effects of CoA, DTNB, CHAPS Detergent and Pre-Incubation Time on the Synthesis Efficiency of Wax Esters (WEs) and Fatty Acid Ethyl Esters (FAEEs) in Arabidopsis Leaf Microsomal Fractions

**Figure 7:**
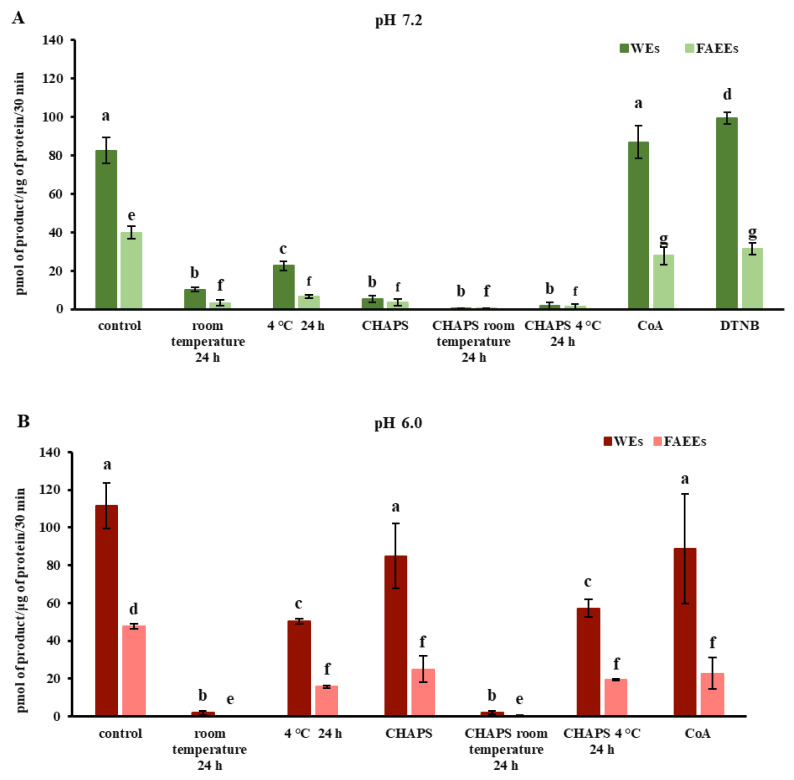
Effect of CoA (0.3 µmol/assay), DTNB (0.7 µmol/assay) and detergent CHAPS (4% solution in the used buffer) on the formation of wax esters (WEs) and fatty acid ethyl esters (FAEEs) in assays with HEPES buffer with pH 7.2 (**A**) and with pH 6.0 (**B**) by enzymes of microsomal fractions from Arabidopsis leaves (containing 13.2 µg of protein). The reactions were carried out for 30 min at 30 °C. Data are presented as the mean ± SD from at least three replicates obtained from one microsomal preparation isolated from 10 to 15 plants. Statistical analysis was performed using one-way ANOVA followed by Tukey’s multiple comparison test. Different letters indicate statistically significant differences (*p* ≤ 0.05).

[^14^C]18:1-FA together with 18:1-OH dissolved in 10 µL of ethanol were used as substrates for the tested enzymes. In assays evaluating the effect of pre-incubation time, the substrates were added at the end of the pre-incubation period The addition of free CoA did not significantly affect the amount of WEs synthesized from the provided substrates at either pH value (7.2 and 6.0). However, in the case of FAEE synthesis, CoA addition reduced the amount of product formed, particularly in assays performed at pH 6.0. The addition of DTNB, which efficiently binds free CoA, slightly inhibited FAEE synthesis and slightly increased WE synthesis (in both cases by approximately 20%), (Figure 7). However, only assays at pH 7.2 were conducted, as DTNB is insoluble at pH 6.0. The lack of stimulation in the synthesis of either type of fatty acids ester in the presence of CoA strongly suggests that the enzymes responsible for esterification do not use acyl-CoA as a substrate.

In the performed assays, 4% CHAPS detergent was used for the solubilization of membrane proteins. In assays with solubilized membrane proteins conducted at pH 7.2, the activities of the tested enzymes were reduced to approximately 5% and 9% of the control values for WE and FAEE synthesis, respectively. However, at pH 6.0, WE synthesis activity was reduced by only about 27%, while FAEE synthesis activity decreased by about 47% compared to the control values (Figure 7).

**Figure 8:**
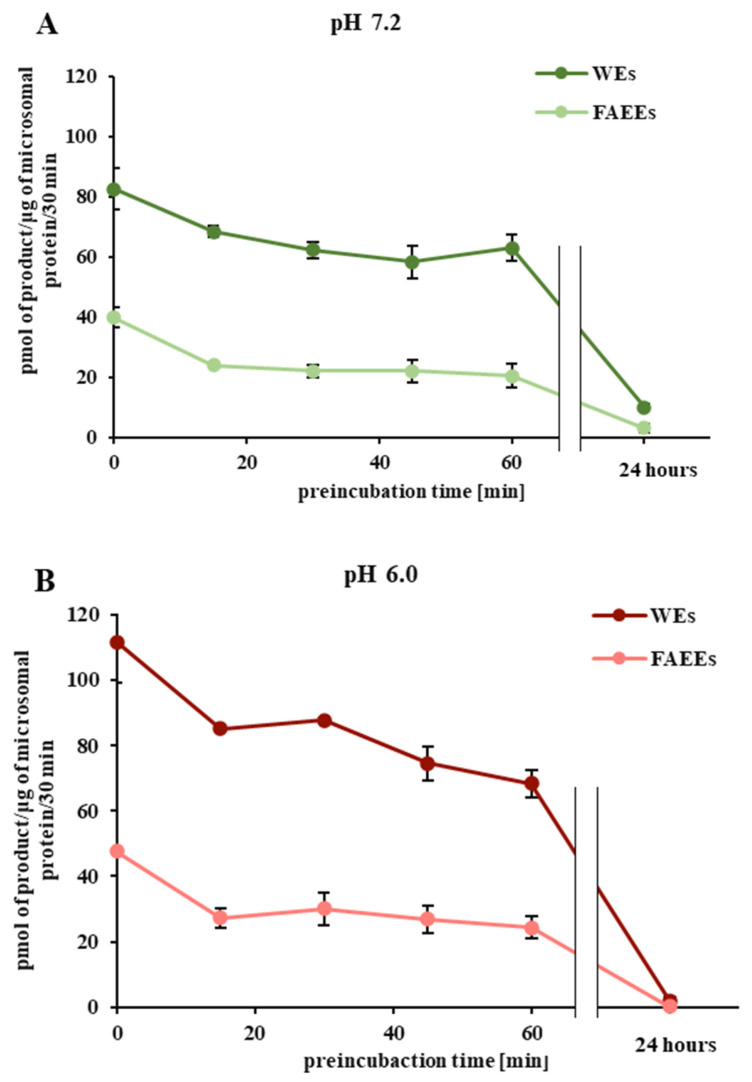
Effect of pre-incubation time at 30 °C of microsomal fractions from Arabidopsis leaves (containing 13.2 µg of protein) on the intensity of formation of wax esters (WEs) and fatty acid ethyl esters (FAEEs) in assays with HEPES buffer with pH 7.2 (**A**) and with pH 6.0 (**B**). The reactions were carried out for 30 min at 30 °C. Data are presented as the mean ± SD from at least three replicates obtained from one microsomal preparation isolated from 10 to 15 plants.

Pre-incubation of solubilized microsomal membrane proteins (in buffer containing CHAPS) for 24 h at room temperature almost completely abolished the activity of the tested enzymes at both pH 7.2 and pH 6.0 (Figure 7). Similarly, pre-incubation of non-solubilized microsomes (not treated with CHAPS) in both incubation buffers (pH 7.2 and pH 6.0) for the same duration reduced enzymatic activity to trace levels, particularly at pH 6.0 (Figure 8). Pre-incubation of these microsomes (not treated with CHAPS) for up to 60 min at 30 °C reduced FAEE synthesis to approximately 51% of activity observed in non- pre-incubated controls at both pH 6.0 and 7.2, while WE synthesis was reduced to about 73% at pH 6.0 and 54% at pH 7.2. The most pronounced decrease in activity was observed during the first 15 min of pre-incubation (Figure 8). However, overnight pre-incubation of these membranes at 4 °C reduced WE synthesis activity to 45% and 27% of control values at pH 6.0 and 7.2, respectively, and almost completely abolished FAEE synthesis activity, which decreased to 33% and 17% of the control activities at pH 6.0 and 7.2, respectively. Overnight pre-incubation of membranes at 4 °C in the presence of CHAPS did not affect the activity of either enzyme at pH 6.0, whereas it completely abolished both WE and FAEE synthesis activities at pH 7.2.

### 2.2. Substrate Specificity of Enzymes from Arabidopsis Leaf Microsomal Fractions Involved in the Synthesis of Wax Esters and of Fatty Acid Ethyl Esters

#### 2.2.1. Utilization of Various Fatty Alcohol for WE Synthesis by Enzymes of Arabidopsis Leaf Microsomal Fractions

The results presented in previous sections concerning WE synthesis by microsomal enzymes from Arabidopsis leaves were obtained using [^14^C]18:1-FA and 18:1-OH as exogenous substrates. In the experiments described here, assays containing [^14^C]18:1-FA in combination with 12 different fatty alcohols were carried out at both pH 6.0 and pH 7.2.

**Figure 9:**
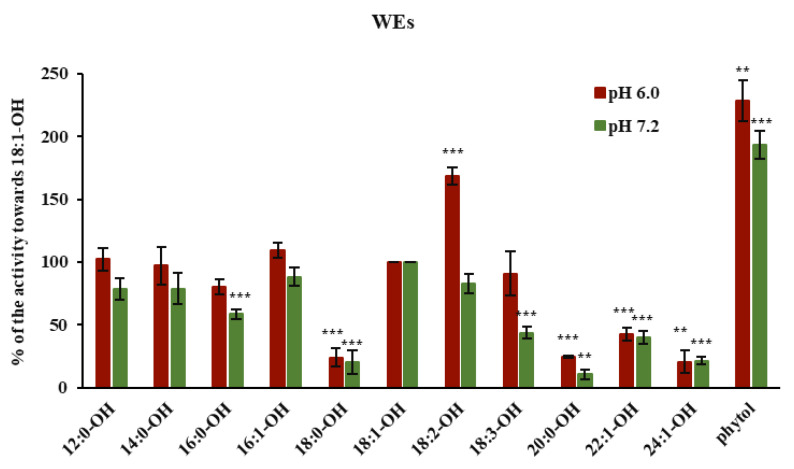
Utilization of various fatty alcohols for wax ester formation by enzymes from microsomal fractions of Arabidopsis leaves (containing 13.2 µg of protein) in HEPES buffer at pH 7.2 and pH 6.0. The reactions were carried out for 30 min at 30 °C. Data are presented as the mean ± SD from at least three replicates obtained from one microsomal preparation isolated from 10 to 15 plants. Statistical significance was determined using a two-tailed Student’s *t*-test comparing assays containing 18:1-OH (control) with assays containing other fatty alcohols. Separate statistical analyses were performed for each pH condition. Asterisks indicate significant differences: ** *p* ≤ 0.01; *** *p* ≤ 0.001.

In assays conducted at pH 6.0, WE synthesis activities similar to those with 18:1-OH were observed for 12:0-OH, 14:0-OH, 16:1-OH, and 18:3-OH. The utilization of long-chain saturated fatty alcohols decreased the rate of WE formation. Assays with 16:0-OH yielded approximately 80% of the amount of synthesized WEs obtained in the reference assays (with 18:1-OH), whereas the use of 18:0-OH and 20:0-OH reduced WE synthesis to about 24% of the reference level. Monounsaturated fatty alcohols with acyl chains longer than 18 carbons were also poorly utilized; in assays with 22:1-OH and 24:1-OH, WE production was reduced to approximately 37% and 20% of the reference level (assays with 18:1-OH), respectively. In contrast, with 18:2-OH, the amount of produced WEs was approximately 68% higher than in the reference assay. Among all tested alcohols, phytol was utilized most efficiently. The phytol ester synthesis was more than twofold higher than that observed with oleyl alcohol (the reference alcohol) (Figure 9).

In assays performed at pH 7.2, phytol was also the fatty alcohol utilized most efficiently for WE synthesis, showing approximately 193% activity relative to the reference alcohol (18:1-OH). Among the remaining 11 tested fatty alcohols, 18:1-OH was utilized most efficiently. An increase in the degree of unsaturation level of 18C fatty alcohols decreased their efficiency as substrates for WE synthesis. Similar to the results obtained at pH 6.0, saturated fatty alcohols with a chain length of 16 carbons or longer were utilized less efficiently than 18:1-OH, especially those containing 18 carbons or more. Very-long-chain monounsaturated fatty alcohols were also utilized less efficiently than 18:1-OH (Figure 9).

#### 2.2.2. Utilization of Various Fatty Acids for WE and FAEE Synthesis by Enzymes in Arabidopsis Leaf Microsomal Fractions

In the performed assays, combinations of 18:1-OH with seven different fatty acids were tested at pH 7.2 and pH 6.0. The efficiency of fatty acid utilization for WE and FAEE synthesis depended not only on the specific fatty acid used but also on the pH of the incubation buffers. The effect of pH was particularly pronounced in the case of WE synthesis.

**Figure 10:**
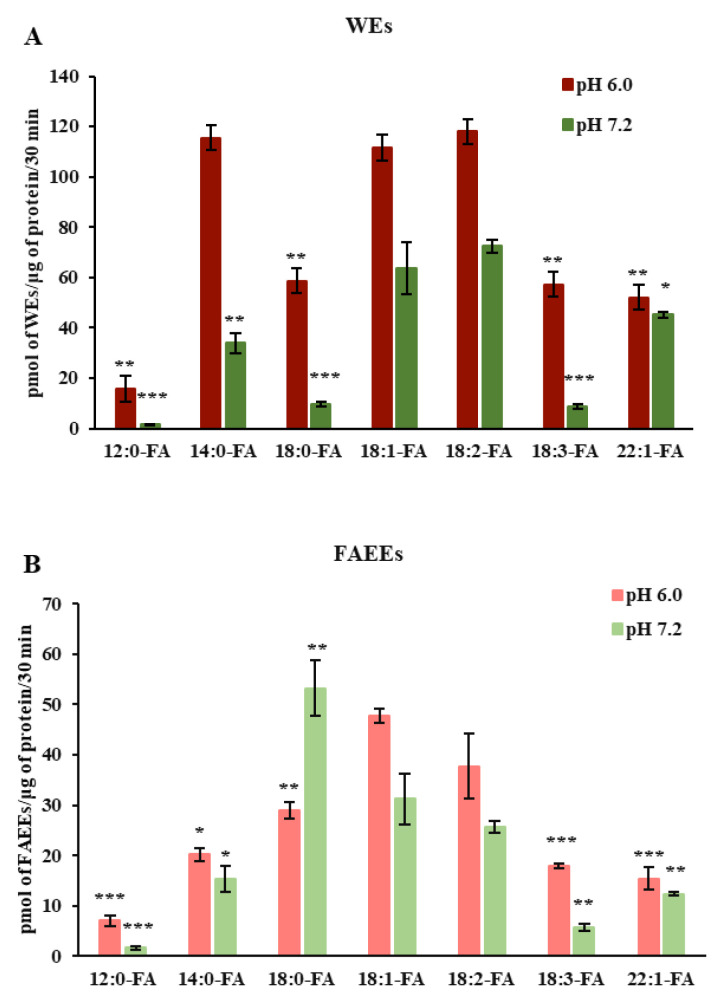
Utilization of various fatty acids for the formation of wax esters (**A**) and fatty acid ethyl esters (**B**) by enzymes from microsomal fractions of Arabidopsis leaves (containing 13.2 µg of protein) in HEPES buffer at pH 7.2 and pH 6.0. The reactions were carried out for 30 min at 30 °C. Data are presented as the mean ± SD from at least three replicates obtained from one microsomal preparation isolated from 10 to 15 plants. Statistical significance was determined using a two-tailed Student’s *t*-test comparing assays containing 18:1-FA (control) with assays containing other fatty acids. Separate statistical analyses were performed for each pH condition. Asterisks indicate significant differences: * *p* ≤ 0.05; ** *p* ≤ 0.01; *** *p* ≤ 0.001.

At pH 6.0, among the seven tested fatty acids, 18:2-FA, 14:0-FA and 18:1-FA were utilized with the highest intensity for WE synthesis by Arabidopsis leaf microsomal enzymes. In contrast 18:0-FA, 18:3-FA and 22:1-FA were utilized at approximately half this efficiency, whereas 12:0-FA was utilized about sevenfold less effectively. At pH 7.2, the overall efficiency of WE synthesis was lower than at pH 6.0 for all tested substrates. The highest rate of WE synthesis at this pH was obtained in assays with 18:2-FA. The use of 18:1-FA resulted in only a slightly lower amount of synthesized WEs (approximately 12% lower). Replacing 18:2-FA with 22:1-FA or 14:0-FA reduced WE formation by approximately 37% and 53%, respectively. The presence of 18:0-FA, 18:3-FA or 12:0-FA in the assays resulted in a significant reduction in WE synthesis—by approximately 87%, 88% and 98%, respectively, compared to assays containing 18:2-FA (Figure 10A).

In assays performed at pH 6.0 for FAEE synthesis, 18:1-FA was the most efficiently utilized substrate, followed by 18:2-FA, 18:0-FA, 14:0-FA, 18:3-FA, 22:1-FA and 12:0-FA, which yielded 79%, 60%, 42%, 37%, 32% and 14%, respectively, of the amount of FAEEs formed in assays containing 18:1-FA. At pH 7.2, 18:0-FA was utilized most efficiently, followed by 18:1-FA, 18:2-FA, 14:0-FA, 22:1-FA, 18:3-FA and 12:0-FA (which showed efficiencies of 59%, 48%, 29%, 23% 11% and 3%, respectively, relative to assays with 18:0-FA) (Figure 10B).

#### 2.2.3. Formation of Esters of Short-Chain Primary Alcohols with Various Fatty Acids by Enzymes from Arabidopsis Leaf Microsomal Fractions

Six different fatty acids (14:0-FA, 18:0-FA, 18:1-FA, 18:2-FA, 18:3-FA and 22:1-FA) were used as exogenous substrates for the studied enzymes, each in combination with four short-chain alcohols (methanol, ethanol, propanol and butanol). The assays were carried out in HEPES buffer at pH 7.2 and pH 6.0.

**Figure 11:**
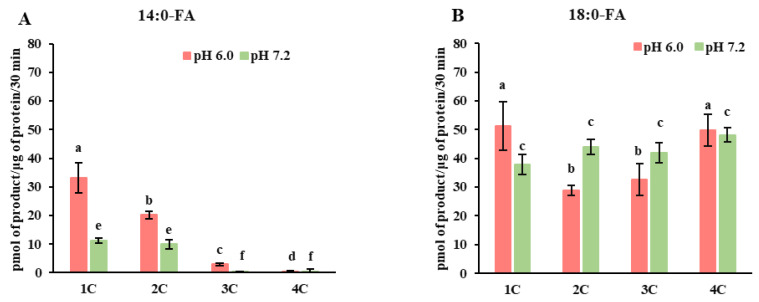

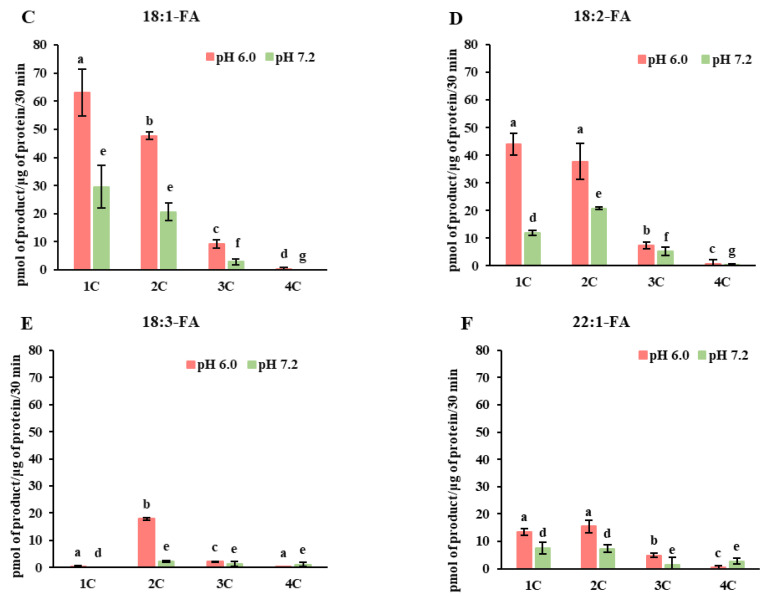
Utilization of short-chain primary alcohols for the synthesis of esters with various fatty acids by enzymes from microsomal fractions of Arabidopsis leaves (containing 13.2 µg of protein) in HEPES buffer at pH 7.2 and pH 6.0. The reactions were carried out for 30 min at 30 °C. Data are presented as the mean ± SD from at least three replicates obtained from one microsomal preparation isolated from 10 to 15 plants. Statistical analysis was performed using one-way ANOVA followed by Tukey’s multiple comparison test. Different letters indicate statistically significant differences (*p* ≤ 0.05). C1—methanol; C2—ethanol; C3—propanol; C4—butanol. (**A**)—assays with 14:0-FA; (**B**)—assays with 18:0-FA; (**C**)—assays with 18:1-FA; (**D**)—assays with 18:2-FA; (**E**)—assays with 18:3-FA; (**F**)—assays with 22:1-FA.

The intensity of ester synthesis from the studied short-chain alcohols and fatty acids depended on the type of alcohol and the specific fatty acid used. The pH of the assays also significantly affected this process. In general, except for assays involving 18:0-FA, ester formation was higher at pH 6.0. When 14:0-FA was used, the amount of synthesized esters was highest in the presence of methanol, followed by ethanol and propanol. In contrast, butanol ester synthesis was close to the detection limit, as was that of propanol esters formed at pH 7.2. In assays with 18:0-FA, the formation of esters with all four tested short-chain alcohols was relatively high and did not differ significantly among the alcohols or between the tested pH values. For 18:1-FA and 18:2-FA, the relative utilization of the tested alcohols was similar to that observed for 14:0-FA, although the total amount of synthesized esters was generally higher. In assays with 18:3-FA, the amount of formed esters was very low (often near the detection limit), except for ethyl ester formation at pH 6.0. The formation of esters from 22:1-FA was also relatively low, although in most cases slightly higher than observed in assays with 18:3-FA (Figure 11).

### 2.3. Activity of Wax Ester and Fatty Acid Ethyl Ester Synthesizing Enzymes in Microsomal Fractions of Arabidopsis Plants Cultivated in Soil and Liquid Culture

In the presented assays, [^14^C]18:1-FA and 18:1-OH dissolved in 10 µL of ethanol were used as exogenous substrates.

**Figure 12:**
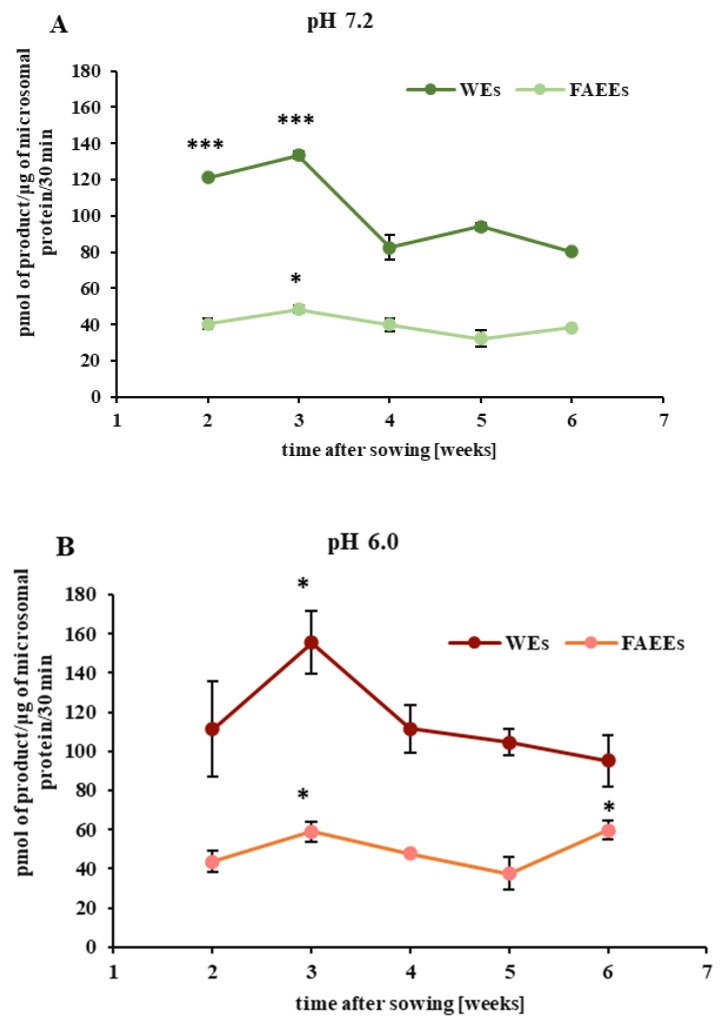
Formation of wax esters (WEs) and fatty acid ethyl esters (FAEEs) in HEPES buffer at pH 7.2 (**A**) and pH 6.0 (**B**) by enzymes from microsomal fractions isolated from Arabidopsis rosette leaves at different developmental stages. Microsomal fractions corresponding to 13.2 µg protein were used in each assay. Reactions were carried out at 30 °C for 30 min. Data are presented as the mean ± SD from at least three replicates obtained from one microsomal preparation isolated from 10 to 15 plants. Statistical significance was determined using a two-tailed Student’s *t*-test comparing samples isolated 4 weeks after sowing (control) with samples isolated at other developmental stages. Asterisks indicate significant differences: * *p* ≤ 0.05; *** *p* ≤ 0.001.

**Figure 13:**
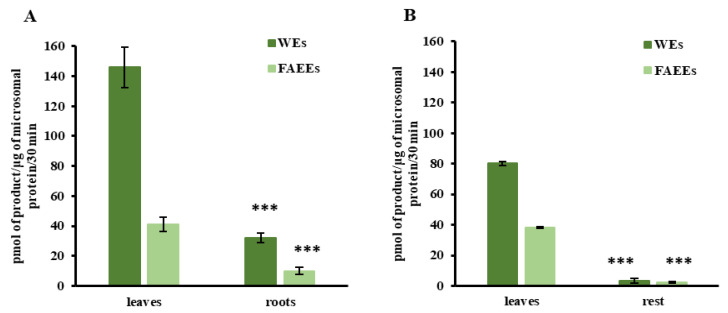

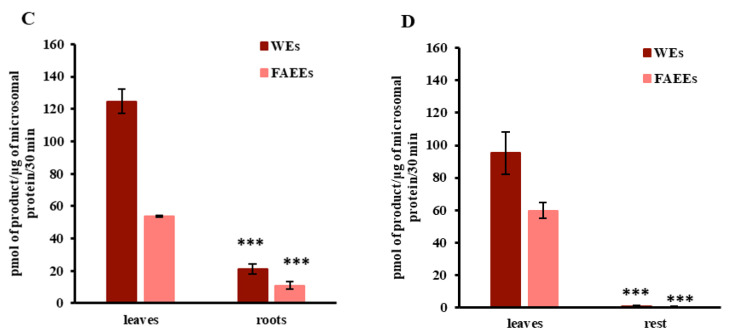
Formation of wax esters (WEs) and fatty acid ethyl esters (FAEEs) in assays with HEPES buffer with pH 7.2 (**A**,**B**) and with pH 6.0 (**C**,**D**) by enzymes of microsomal fractions isolated from leaves and roots of Arabidopsis plants cultivated in liquid culture (**A**,**C**) and from Arabidopsis rosettes leaves and “rest” of Arabidopsis plants (shoots, flowers and cauline leaves) from six-week-old plants cultivated in soil (**B**,**D**). Amounts of microsomal fractions equal to 13.2 µg of protein were added. The reactions were carried out for 30 min at 30 °C. Data are presented as the mean ± SD from at least three replicates obtained from one microsomal preparation isolated from 10 to 15 plants. Statistical significance was determined using a two-tailed Student’s *t*-test comparing samples isolated from leaves (control) with samples isolated from roots or the remaining aerial tissues. Asterisks indicate significant differences: *** *p* ≤ 0.001.

In assays using microsomal fractions prepared from Arabidopsis rosette leaves collected from 2 to 6 weeks old plants, the highest activities of both WE and FAEE synthesis were recorded in 3-week-old plants. Enzymatic activities were lower in microsomal fractions obtained from both younger and older plants, particularly regarding WE synthesis. Similar trends were observed at both pH 6.0 and pH 7.2 (Figure 12). In six-week-old plants, the tested enzymatic activities were significantly lower in microsomal fractions prepared from shoots, flowers and cauline leaves (“rest”) than in those from rosette leaves, especially at pH 6.0 (Figure 13). In assays with microsomes from Arabidopsis plants grown in liquid culture, the WE synthesis activity was 4.5- and 5.9-times higher in leaves than in roots at pH 7.2 and 6.0, respectively. Similarly, FAEE synthesis activity was 4.1- and 4.9-times higher in leaf microsomal fractions than in those from roots (Figure 13).

## 3. Discussion

The esterification reactions of free fatty acids with free primary alcohols carried out by enzymes of microsomal fractions of Arabidopsis leaves and roots have been confirmed by the performed studies. Esterification of free fatty acids with free fatty alcohols by enzymes of acetone powder from broccoli leaves was already reported in the late 1960s [19]. However, partial characterization of the enzymes catalyzing the reactions was not carried out until the beginning of the 21st century [20,21]. In those studies, it was suggested that the synthesis of WE and FAEE synthesis could be catalyzed by separate enzymes. It was also proposed that both enzymes contain a lipase motif in their structure, as both activities were strongly inhibited by tetrahydrolipstatin, a well-known lipase inhibitor [20,21]. The biochemical characterization of enzymes able to esterify free fatty acids with free alcohols, as performed in present study, does not provide a clear answer as to whether WEs and FAEEs are synthesized by different enzymes, since in many cases environmental factors regulate both activities in a similar manner. However, certain differences were also noted; thus, we cannot exclude that separate enzymes catalyze these two activities. The obtained results further suggest that among the enzymes responsible for esterification of free fatty acids, isoforms exhibiting the highest activity at two distinct pH values—i.e., pH 6.0 and pH 7.2—may be present. These putative isoforms appeared to differ in their biochemical properties including temperature dependence, responses to divalent cations, or stability after microsomal membrane solubilization, as well as substrate specificity towards both free fatty acids and free alcohols. The suggested activities—corresponding to pH 6.0 and pH 7.2—were both inhibited by tetrahydrolipstatin (which may indicate their lipase-like nature; however, their responses to this inhibitor differed slightly. Both hypothetical isoforms were relatively stable at 30 °C for at least 60 min, but they lost their activity after 24 h of incubation at room temperature; thus, in this regard, they do not significantly differ from each other. However, after membrane solubilization with 4% CHAPS, the activity associated with the potential pH 6.0 isoform was largely retained, whereas the activity at pH 7.2 showed a significant decline. Thus, it seems that at least the former potential isoforms could be purified, at least partially, in future studies.

The tested enzymes were present in the microsomal fractions of rosette leaves and other parts of Arabidopsis plants grown in soil. They were also active in microsomal fractions from the leaves and roots of Arabidopsis plants cultivated in liquid cultures. Among the tested microsomal fractions, the highest activity was found in microsomes from leaves, whereas the lowest activity was in microsomes prepared from stems with cauline leaves and flowers (other parts). We did not test the activity of the studied enzymes in microsomes isolated from the roots of plants grown in soil; however, in plants cultivated in liquid culture, both types of activity were higher in microsomes from leaves. In previous studies [20] using microsomal fractions from the leaves and roots of Arabidopsis plants grown in liquid culture, the ability to synthesize WEs and FAEEs was similar to that obtained in the present research; however, the differences in WE synthesis capacity between these organs were much higher. The age of the leaves also affects the activity of the tested enzymes, with the highest activity observed in three-week-old plants. Rosette leaves of both younger and older plants were characterized by lower enzymatic activity, particularly with respect to WE synthesis. So far, there is no information available on the activity of the studied enzymes in the leaves of other plant species. However, the tested enzymatic activities were studied in different parts of wheat roots cultivated in liquid culture [21]. In control plants, both the ability to synthesize WEs and the ability to synthesize FAEEs (from free fatty acids and free alcohols) were much higher in older root regions than in approximately 1 cm long root tips containing meristematic and elongation-zone cells. These enzymatic activities were strongly enhanced by alloxydim, a grass herbicide. The ability to synthesize WEs increased only in root tips, but the ability to synthesize FAEEs also increased in older parts of the roots. To date, it is not known whether the increase in the activity of the tested enzymes was associated with changes in the tissue structure of the root tips, whose growth was inhibited, or whether the increased ability to convert free fatty acids into ester forms was a type of defense mechanism against stress caused by herbicide treatment. Currently, there is no information available on whether the fermentation process is intensified and ethanol production is enhanced in grasses treated with herbicides. However, it is well known that different types of stress increase non-oxidative respiration [25]. Furthermore, it has been shown that wheat roots treated with the herbicide alloxydim exhibit a considerable increase in phospholipid acyl-hydrolase activity [21]. Thus, this type of stress triggers the release of free fatty acids in treated roots. Consequently, the increased activity of enzymes responsible for the esterification of free fatty acids could represent a defense mechanism. Other environmental stresses also increase free fatty acid levels in plants subjected to stress [26,27,28]. Free fatty acids are negatively charged; therefore, their presence in membranes could affect, for example, the membrane electrical potential. It is therefore possible to speculate that the studied membrane-bound enzymes are involved in a protective mechanism against the effects of free fatty acids on membrane properties. The esterification of free fatty acids into ester form eliminates their impact on membrane potential. Moreover, the stress-associated increase in fermentation could provide a source of ethanol required for such esterification. However, apart from the effect of the herbicide alloxydim on the activity of the tested enzymes in stressed plants, no information is currently available regarding the influence of other stress factors. Therefore, this issue remains an important area for further study.

Contrary to the possibility of ethanol production in various plant tissues, information regarding the synthesis of long-chain alcohols in plant cells—apart from those involved in epicuticular wax and suberin synthesis—is rather scarce. These alcohols are, however, synthesized primary in epidermal and endodermal cells [1]. Thus, the high activity of enzymes responsible for wax ester formation in microsomal fractions prepared from whole leaves or roots is somewhat surprising. Among the tested long-chain alcohols, however, these enzymes exhibited the highest activity towards phytol. This isoprenoid of alcohol may be present not only in chloroplasts, where it is a component of chlorophyll, but also in other parts of the cell. During chlorophyll breakdown, phytol is released and can likely be transported outside the chloroplast. Free phytol exhibits toxic effects on membranes; therefore, its conversion into phytol fatty acid esters protects the membrane integrity [29]. The high activity of the tested enzymes in leaves is consistent with their potential role in converting free phytol into its storage form.

Information regarding WEs and FAEEs in plants is presented in Section 1. WEs are usually associated with the cuticular wax layer, whereas FAEEs have been identified in only a few plant species and in organs such as pollen, fruit arils, and leaves, as well as in some plant oils. Thus, this information is rather limited and does not include different cellular compartments, such as cellular membranes, where the tested enzymes involved in the synthesis of these esters are localized. Apart from the effect of the herbicide alloxydim on the activity of the tested enzymes, no other information is currently available regarding the impact of other stress factors. There is also a lack of information concerning the widespread occurrence of these enzymes throughout the plant kingdom. Thus, the present study, together with two earlier reports [20,21], opens a new and still underexplored area of lipid metabolism related to the protection of membrane integrity against the harmful effects of an excess free fatty acids and/or free fatty alcohols within membrane structures.

## 4. Materials and Methods

### 4.1. Reagents

[^14^C]-labeled fatty acids were purchased from PerkinElmer Life Science (Waltham, MA, USA). Other chemicals used for analysis were obtained from Sigma-Aldrich (St. Louis, MO, USA), Merck (Darmstadt, Germany) or Larodan Fine Chemicals (Malmö, Sweden).

### 4.2. Plant Material

Analyses were performed using *Arabidopsis thaliana* (ecotype Columbia-0). Plants were harvested at 2, 3, 4 (0 DAF—0 days after flowering), 5 and 6 weeks of age. Rosette leaves from freshly harvested plants were used to isolation of microsomal fractions. For the six-week-old plants, microsomal fractions were also prepared from shoots, flowers and cauline (non-rosette) leaves.

Additionally, microsomal fractions were isolated from the roots and leaves of *A. thaliana* (ecotype Columbia-0) grown for four weeks in in vitro culture. Initially, plants were grown for two weeks on a solid medium (1/3 MS [30], 1% sucrose, and 0.8% agar). Subsequently, they were transferred to the liquid medium (1/2 MS [30] and 1% sucrose) for an additional two weeks.

Prior to sowing on solid medium, seeds were surface-sterilized by washing with 3% hypochlorite for 15 min, followed by a 1 min wash with 70% ethanol. The seeds were then rinsed four times with distilled water. All plants were maintained in a growth chamber at a constant temperature of 21 °C and 60% relative humidity, under a long-day photoperiod (16 h light/8 h dark) with a light intensity of 120 µmol photons m^−2^ s^−1^.

### 4.3. Microsomal Membrane Preparation

The collected plant material was placed in a glass homogenizer and ground in 0.1 M potassium phosphate buffer (pH 7.2) containing bovine serum albumin (1 mg/mL), sucrose (0.33 M), and catalase (1000 U/mL). The homogenates were filtered through two layers of Miracloth, diluted with fresh extraction buffer to 27 mL, and centrifuged at 20,000× *g* for 12 min. The obtained supernatants were again filtered through two layers of Miracloth and centrifuged at 100,000× *g* for 90 min. The resulting pellets, representing the microsomal fractions, were washed with 0.1 M potassium phosphate buffer (pH 7.2) and resuspended in a small volume of the same buffer. All stages of microsomal membrane preparation were conducted at 4 °C, and the isolated fractions were stored at −80 °C until further analysis. Protein concentrations in the microsomal fractions were determined using the Pierce™ BCA Protein Assay Kit according to the manufacturer’s instructions (Pierce Chemical, Dallas, TX, USA).

### 4.4. Enzyme Assays

#### 4.4.1. Optimization of Enzyme Assays

Optimization tests were carried out to establish the optimal conditions for enzyme assays used to determine the activity and substrate specificity of the enzymes from *A. thaliana* rosette leaves. Five factors were analyzed: the amount of microsomal fraction, reaction time, temperature, buffer pH, and the addition of 10 mM of selected ions (K^+^, Ca^2+^, and Mg^2+^). In all of these assays, 19 nmol of 18:1-OH and 5 nmol of [^14^C]18:1-FA were used as exogenous substrates. The substrates were added in 10 µL ethanol to crude microsomal fractions resuspended in 90 μL of 50 mM HEPES buffer (pH 7.2 or pH 6.0, as indicated in the figures). The results of these optimization experiments were used for further analyses. Microsomal fractions isolated from *A. thaliana* rosette leaves harvested at 0 DAF were used in all optimization experiments.

#### 4.4.2. Enzyme Stability and Substrate Specificity

Enzyme stability was assessed by incubating crude microsomal fractions in 90 µL of 50 mM HEPES buffer (pH 7.2 or 6.0) at 30 °C for the durations indicated in the figures. Following incubation, exogenous substrates ([^14^C]18:1-FA and 18:1-OH dissolved in 10 µL of ethanol) were added.

Substrate specificity towards fatty acids was subsequently analyzed using the following [^14^C]-labeled fatty acids: [^14^C]12:0-FA (lauric acid), [^14^C]14:0-FA (myristic acid), [^14^C]18:0-FA (stearic acid), [^14^C]18:1-FA (oleic acid), [^14^C]18:2-FA (linoleic acid), [^14^C]18:3-FA (linolenic acid), and [^14^C]22:1-FA (erucic acid). Reaction mixtures contained 19 nmol of exogenous 18:1-OH, 5 nmol of the respective [^14^C]-fatty acid, 10 µL of ethanol, and microsomal fraction aliquots corresponding to 13.2 µg of protein in 90 µL of 50 mM HEPES buffer (pH 7.2 or 6.0).

Substrate specificity towards alcohols was also tested using the following long-chain alcohols: 12:0-OH (lauryl), 14:0-OH (myristyl), 16:0-OH (cetyl), 16:1-OH (palmitoleyl), 18:0-OH (stearyl), 18:1-OH (oleyl), 18:2-OH (linoleyl), 18:3-OH (linolenyl), 20:0-OH (arachidyl), 22:1-OH (erucyl), 24:1-OH (nervonyl), and phytol. These reaction mixtures contained 5 nmol of the respective alcohol, 5 nmol of [^14^C]18:1-FA, 10 µL of ethanol, and 13.2 µg of microsomal protein. For short-chain alcohols (methanol, ethanol, 1-propanol, and 1-butanol), the reaction mixtures contained 5 nmol of the respective [^14^C]-fatty acid and 10 µL of the alcohol, with microsomal protein as described above.

ATP, CoA, DTNB, and CHAPS were dissolved in 50 mM HEPES buffer (pH 7.2 or 6.0) at the following concentrations per assay: 0.3 µmol ATP, 0.3 µmol CoA, 0.7 µmol DTNB, and 4% CHAPS. Since DTNB is insoluble at pH 6.0, assays involving DTNB were performed only at pH 7.2.

Unless stated otherwise, microsomal fractions from *A. thaliana* rosette leaves harvested at 0 DAF were used. All reactions were carried out at 30 °C for 30 min with shaking (1250 rpm) and terminated by adding 375 μL of chloroform:methanol (1:2, *v*:*v*), 125 µL of 0.15 M acetic acid, and 125 μL of chloroform. After vigorous shaking and centrifugation, the chloroform fractions were collected. Extracted lipids were separated by thin-layer chromatography on silica gel 60 plates (Merck) using a hexane:diethyl ether:glacial acetic acid (70:30:1, *v*:*v*:*v*) solvent system. Reaction products ([^14^C]-wax esters and [^14^C]-fatty acid ethyl esters) were visualized and quantified using electronic autoradiography (Instant Imager; Canberra Packard, Schwadorf, Austria).

### 4.5. Statistical Analysis

All experiments were performed using three independent replicates, each representing one microsomal preparation isolated from 10 to 15 plants. Data are presented as the mean ± standard deviation (SD). Statistical analyses were performed using either a two-tailed Student’s *t*-test or one-way analysis of variance (ANOVA) followed by Tukey’s multiple comparison test, as indicated in the corresponding figure legends. Student’s *t*-test was applied for pairwise comparisons between control and treatment groups, whereas one-way ANOVA followed by Tukey’s post hoc test was used for comparisons involving more than two experimental groups. Separate statistical analyses were performed for each pH condition when applicable. Differences were considered statistically significant at *p* ≤ 0.05.

## 5. Conclusions

In conclusion, this study provides a comprehensive biochemical characterization of the fatty acid esterification pathways occurring within the microsomal fractions of *Arabidopsis thaliana* leaves. Our findings demonstrate that the microsomal membrane environment supports the synthesis of both wax esters (WEs) and fatty acid ethyl esters (FAEEs), exhibiting two distinct pH optima at 6.0 and 7.2. While these dual optima display distinct sensitivities to temperature, detergent solubilization (CHAPS), and divalent cations, their closely shared substrate preferences, particularly their high affinity for phytol, and identical developmental and tissue-specific profiles strongly suggest that these apparent activities represent either tightly regulated physiological states of a single multi-functional enzymatic system or closely related membrane-bound proteins. Furthermore, the strong sensitivity of these esterification activities to tetrahydrolipstatin highlights their potential lipase-like catalytic nature. Ultimately, the capacity of these microsomal activities to efficiently utilize free fatty acids, short/long-chain alcohols, and free phytol supports the hypothesis that these pathways may serve as a vital protective mechanism within plant membranes, potentially acting to detoxify excess lipophilic molecules under physiological transitions or environmental stress.

## Data Availability

The original contributions presented in this study are included in the article. Further inquiries can be directed to the corresponding author.
